# Removal of Heavy Metal Ions from One- and Two-Component Solutions via Adsorption on N-Doped Activated Carbon

**DOI:** 10.3390/ma14227045

**Published:** 2021-11-20

**Authors:** Justyna Kazmierczak-Razna, Anetta Zioła-Frankowska, Piotr Nowicki, Marcin Frankowski, Robert Wolski, Robert Pietrzak

**Affiliations:** 1 Łukasiewicz Research Network-Institute of Non-Ferrous Metals, Division in Poznań, Central Laboratory of Batteries and Cells, Forteczna 12, 61-362 Poznań, Poland; justyna.kazmierczak-razna@claio.poznan.pl; 2 Faculty of Chemistry, Adam Mickiewicz University in Poznań, 61-614 Poznań, Poland; anettazf@amu.edu.pl (A.Z.-F.); marcin.frankowski@amu.edu.pl (M.F.); wola@amu.edu.pl (R.W.); pietrob@amu.edu.pl (R.P.)

**Keywords:** brown coal, activated carbons, chemical activation, ammoxidation, heavy metals, simultaneous adsorption

## Abstract

This paper deals with the adsorption of heavy metal ions (Cu^2+^ and Zn^2+^) on the carbonaceous materials obtained by chemical activation and ammoxidation of Polish brown coal. The effects of phase contact time, initial metal ion concentration, solution pH, and temperature, as well as the presence of competitive ions in solution, on the adsorption capacity of activated carbons were examined. It has been shown that the sample modified by introduction of nitrogen functional groups into carbon structure exhibits a greater ability to uptake heavy metals than unmodified activated carbon. It has also been found that the adsorption capacity increases with the increasing initial concentration of the solution and the phase contact time. The maximum adsorption was found at pH = 8.0 for Cu(II) and pH = 6.0 for Zn(II). For all samples, better fit to the experimental data was obtained with a Langmuir isotherm than a Freundlich one. A better fit of the kinetic data was achieved using the pseudo-second order model.

## 1. Introduction

It is known that human activity generates large amounts of pollutants, contributing to degradation of the natural environment. A group of particularly troublesome pollutants are metal ions that enter the natural environment, e.g., in exhaust gases after fuel combustion, residues of catalysts, and in fertilizers. Particularly dangerous among them are heavy metal ions including lead, zinc, copper, cadmium, mercury, cobalt, nickel, and so on, polluting the soil and water environment. The health risk related to their presence is a consequence of their high toxicity and capability of accumulation [1,2]. The content of heavy metal ions in wastewater, drinking water, and water for industrial applications is under continuous monitoring. The most popular methods for the removal of heavy metal ions from aqueous media are chemical precipitation, reversed osmosis, evaporation, and ion exchange [3,4]. These methods have many advantages, although often they prove little effective, especially at low concentrations of heavy metals, they also generate toxic deposits and are rather expensive [5]. An alternative to the above methods can be sorption on activated carbons.

Activated carbons are universal sorbents used in many areas of research and technology as well as in everyday life. Their very good sorption abilities are mainly related to their large surface area; strongly developed microporous structure; and the presence of numerous functional groups containing heteroatoms such as nitrogen, oxygen, or sulfur on their surface. Thanks to the above properties, activated carbons have been successfully used for the removal of pollutants from water and air; for separation of gas mixtures; and for recovery of valuable substances, e.g., gold or silver. Adsorption on activated carbons has been applied in many sectors of the economy in a wide range of processes from food, pharmaceutical, and petrochemical industries to purification of drinking water, post-industrial wastewater, municipal wastewater, and exhaust gases [6,7,8,9].

Modified activated carbons are of particular importance in this respect, because of a very wide gamut of their potential applications. Carbonaceous materials enriched in oxygen, sulfur, phosphorus, or nitrogen containing functional groups may be applied for the adsorption of gases of acidic character such as H_2_S and NO_x_, as well as for adsorption of metal ions, dyes, aromatic and aliphatic amines, or phenol from the liquid phase. For example, S-doped activated carbons are very effective in the removal of lead, cadmium, and mercury ions from drinking and wastewater. Phosphorus-enriched carbon materials show excellent cation-exchanging properties. In turn, nitrogen-doped activated carbons are very effective in H_2_S, SO_2_, CO_2_, NO_2_, or VOCs’ removal from gas stream as well as in the removal of copper and lead ions, amines, phenol, and its derivatives’ adsorption from aqueous solutions [10,11,12,13,14].

Sorption of metal ions, e.g., (Cu(II), Zn(II), Pb(II), Cd(II), Mn(II), Co(II), Ni(II), Fe(III), As(III), or Bi(III)), on activated carbons modified in a different way is quite often described in the literature on the subject; however, it most often concerns their removal from one-component systems. Taking that into account, the main aim of this study was to evaluate the effectiveness of sorption of selected heavy metals (Cu^2+^ and Zn^2+^) not only from single component systems, but also from binary solutions on the activated carbons obtained by chemical activation of low quality brown coal (unfit for use for energy purposes) with KOH and enriched in nitrogen in the process of ammoxidation (simultaneous oxidation and nitrogenation).

## 2. Materials and Methods

### 2.1. Materials

Sorption studies were carried out using the carbonaceous sorbents prepared by chemical activation with KOH (A) or activation with KOH followed by ammoxidation process (AN). The detailed sample preparation, detailed procedure of their carbonization, activation, nitrogen incorporation into carbon structure, as well as physicochemical characterization are given in our earlier work [15]. Briefly, demineralized brown coal (of grain size ≤ 0.2 mm) was firstly subjected to the carbonization process, followed by chemical activation with KOH in an argon atmosphere at 700 °C. The last step of the thermo-chemical treatment was nitrogen incorporation via the ammoxidation process, conducted at 350 °C. The final products are highly microporous activated carbons with a very well-developed surface area of 2156 and 1877 m^2^/g, respectively. The prepared carbon materials also differ in terms of the acid–base nature of the surface as well as nitrogen content. The product of chemical activation (A) shows an acidic character of the surface and very low nitrogen contribution (0.4 at. %) in the form of pyrrolic, pyridonic (N−5), as well as pyridine N-oxide groups. According to the XPS study (described in details in the previous work [16]), the N-doped activated carbon (AN) is characterized by a high nitrogen content (bulk content 5.8 at. %, surface content 10.2 at. %), mainly in the form of amines, amides, nitriles, as well as imines and lactams. Moreover, basic functional groups predominate on its surface [15]. Morphologies of the samples (before and after metal ions’ adsorption) were analyzed on the basis of scanning electron microscope images (PHILIPS, Eindhoven, Netherlands) recorded under the following conditions: working distance of 14 mm, accelerating voltage of 15 kV, and digital image recording by DISS. The prepared materials were also characterised by X-ray diffraction (XRD) using a D8 Advance diffractometer (Bruker, Billerica, MA, USA) equipped with Johansson monochromator (CuKα, α = 0.154 nm) and silicon strip detector LynxEye, with a step size of 0.05° in the high-angle range. IRSpirit Fourier transform infrared spectrophotometer with a QATR™-S single-reflection ATR measurement accessory (Shimadzu, Nishinokyo Kuwabara-cho, Kyoto, Japan) was employed to measure changes in surface functional groups before and after adsorption.

The salts Cu(NO_3_)_2_·2H_2_O and Zn(NO_3_)_2_·4H_2_O were used as sources of metal ions. The stock solutions were obtained by dissolving these salts in distilled water using 1 L flasks and stored in a fridge. All reagents were of analytical grade and purchased from Avantor Performance Materials Poland S.A. (Gliwice, Poland).

### 2.2. Kinetic Studies

Stock solutions (1000 mg/L) of Cu(II) or Zn(II) and both Zn(II) and Cu(II) were prepared by dissolving the appropriate masses of their salts, Cu(NO_3_)_2_·2H_2_O and Zn(NO_3_)_2_·4H_2_O, in distilled water. In order to obtain solutions of concentrations of 10 mg/L, the stock solutions were diluted. The carbon samples of 0.01 g were placed in 100 mL conical flasks. Then, 50 mL of a metal salt solution was added and shaken mechanically at 180 rpm, at a temperature of 22 °C ± 1 °C, at the phases contact time from 1 to 360 min, at pH 6. After shaking, the solution was filtered off. The content of metal ions was determined by the technique of atomic absorption spectrometry with acetylene-air flame atomization (F-AAS) using an AA7000 spectrometer provided by Shimadzu (Nishinokyo Kuwabara-cho, Kyoto, Japan), equipped with a ASC 7000 autosampler. Determination of metals was performed in three replications with relative standard deviation not exceeding 7%. The reagents used were analytically pure and water was deionized to a resistivity of >18 MΩ in a Milli Q-Direct 8 apparatus (Millipore SAS, Molsheim, France). Standard solutions were made from Merck commercial standards for AAS of a concentration of 1000 mg L−1 (Merck, Darmstadt, Germany). The measured parameters are given in Table 1.

### 2.3. The Effect of pH

The pH effect on metal(II) ions’ sorption was studied for A and AN. A portion of 0.01 g of the sample was shaken with a 25 mL solution of Cu(II), Zn(II), and both Cu(II) and Zn(II) in the concentration of 10 mg/L for 240 min, at pH in the range 2.0–8.0. After shaking, the suspension was filtered off and the final concentration of metals was determined. The solution pH before the experiments was adjusted by adding appropriate amounts of 0.1M HCl and/or 0.1M NaOH. The pH was measured using a pH meter manufactured by Metrohm Ion Analysis (Herisau, Switzerland) equipped with an Unitrode Pt1000 (combined glass pH electrode with a temperature sensor), calibrated with standards solutions of pH 3, 7, and 10.

### 2.4. Adsorption Tests

The equilibrium adsorption isotherms were recorded for 25 mL solutions of Cu(II) or Zn(II) or both Cu(II) and Zn(II) in concentrations in the range 10–50 mg/L, added to 0.01 g of A and AN samples (shaking speed 180, shaking time 360 min, temperature 22 °C ± 1 °C, pH 5). Then, each solution was filtered off and the final concentration of heavy metal ions was determined as described above.

### 2.5. Data Evaluation

The adsorbed amount *q_t_* (mg/g) was calculated from the equation:

(1) $$q_{t}=\frac{\left(C_{0}-C_{t}\right)}{m}\cdot V,$$

where *m* (g) is the mass of sorbent, *V* is the volume of solution (L), and *C*_0_ is the initial concentration of heavy metal ions (mg/L) and *C_t_* is the concentration of heavy metal ions after time *t* (mg/L).

The mechanism of heavy metal ions’ sorption on carbonaceous sorbents was investigated using the pseudo-first-order equation (PFO) proposed by Lagergren, Equation (2) [17]; the pseudo-second-order equation (PSO) proposed by Ho and McKay, Equation (3) [18]; and the intraparticle diffusion model (IPD) used by Webber and Morris, Equation (4) [19], which can be presented as follows:

(2) $$log\left(q_{1}-q_{t}\right)=log\left(q_{1}\right)-\frac{k_{1}\cdot t}{2.303},$$

(3) $$\frac{t}{q_{t}}=\frac{1}{k_{2}\cdot q_{2}^{2}}+\frac{t}{q_{2}},$$

(4) $$q_{t}=k_{i}\cdot t^{\frac{1}{2}}+C,$$

where *k*_1_ is the pseudo-first-order rate constant (1/min), *k*_2_ is the rate constant of the pseudo-second-order sorption (g/(mg∙min)), *k_i_* is the intraparticle diffusion rate constant (mg/g min^1/2^), and *C* is the intercept representing the boundary layer effect.

In order to investigate the heavy metal ions’ adsorption equilibrium on carbonaceous materials, the Langmuir isotherm, Equation (5), and the Freundlich isotherm, Equation (6) [20,21,22,23], were applied. The Langmuir model is expressed as follows [20]:

(5) $$q_{e}=\frac{q_{0}\cdot K_{L}\cdot C_{e}}{1+K_{L}\cdot C_{e}},$$

The Freundlich model is as follows [21]:

(6) qe=KF·Ce1n,

where *C_e_* (mg/L) is the equilibrium concentration of heavy metal ions’ solution, *q_e_* (mg/g) is the amount of metal ions adsorbed at equilibrium, the constant *q*_0_ (mg/g) is the maximum adsorption capacity, and *K_L_* (L/mg) are the characteristics of the Langmuir isotherm. Adsorption parameters were determined on the basis of the linear relationship of *C_e_/q_e_* and *C*_e_. *K_F_* (mg/g·(L/mg)^1/*n*^) is the adsorption capacity characteristic of the Freundlich model, 1/*n* is the Freundlich constant connected with the surface heterogeneity. The parameters *K_F_* and *n* were calculated from the linear relationship of *log C_e_* and *log q_e_*.

## 3. Results

### 3.1. Dynamics and Kinetics of the Metal Adsorption Process

**Figure 1:**
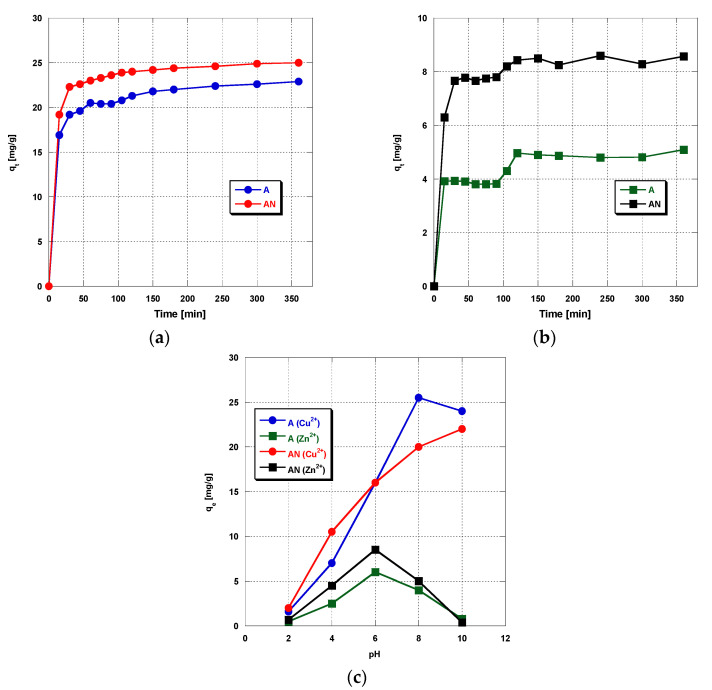
The effect of the phase contact time on the heavy metals ions’ adsorption on activated carbons (**a**) Cu(II) ions’ adsorption, (**b**) Zn(II) ions’ adsorption, and (**c**) simultaneous adsorption of Zn(II) and Cu(II); C_init_ 10 mg/L, pH 6, mass of sorbent: 0.01 g, time 360 min, shaking speed 180 rpm, temperature 22 ± 2 °C.

The first stage of the copper and zinc adsorption study was to check the effect of the time of the process on the amount of metal ions adsorbed from water solutions. The experiments were performed until reaching equilibrium between the adsorbent and metal ions remaining in solution. Figure 1a–c presents the amount of adsorbed metal ions versus the adsorption duration.

For the single-component solution of copper(II) ions, the equilibrium is reached in the first 60 min of shaking (Figure 1a). For the single-component zinc(II) solution and binary solution containing copper(II) and zinc(II) ions, the equilibrium is reached after about 120 min of the process (Figure 1b,c). Introduction of 6 wt.% of nitrogen into the activated carbon structure in the process of ammoxidation had no significant effect on the adsorption of any of the metal ions studied. The rate of sorption of the metal ions studied was different in two stages of the process. In the first stage, below 20 min, a rapid sorption of metals was observed, interpreted as related to the presence of acidic functional groups on the carbon surfaces, which attracted the metal ions endowed with positive change, so at this stage, irreversible chemical adsorption took place. An extension of the time of the process above 20 min caused a small increase in the amount of adsorbed metal ions, until equilibrium. As in this stage, adsorption was fast, a large amount of ions were removed from the solution. In the second stage, the adsorption was slower, most probably because of the saturation of the active centers on the carbon sorbent surface and longer time of metal ions’ diffusion to the deeper layers of the carbon porous structure. On the basis of the data from the sorption tests, the kinetics of adsorption of the metal ions on the carbons studied was characterized. Knowledge of the adsorption mechanisms and kinetics of this process permits the regulation of certain parameters to achieve the highest effectiveness of metal ions’ removal. For this reason, the three kinetic models were considered: the pseudo-first-order, the pseudo-second-order, and the intraparticle diffusion models, in order to determine the kinetics of heavy metals’ adsorption on the activated carbons.

The kinetic parameters are listed in Table 2, Table 3 and Table 4. The experimental data obtained for sorption of copper(II) ions from the single-component solution are best described by the kinetic equation of pseudo-second-order. The square of the correlation coefficient of the linear regression equations for this type of kinetics was 0.9991–0.9997, much higher than that obtained for the pseudo-first-order kinetics or that for the intraparticle diffusion model. In view of the literature data [24], it is reasonable to assume that the high correlation coefficients for the PSO kinetic equation suggest chemisorption of Cu^2+^, which is why the rate of adsorption was dependent on the valence or valence forces.

Moreover, for sorption of zinc(II) ions from the single-component solution, the highest correlation coefficients R^2^ of 0.9927–0.9989 were obtained for the PSO kinetic equation. Similar results were obtained for the simultaneous adsorption of Cu^2+^ and Zn^2+^ from binary solutions. The correlation coefficient R^2^ was the highest for PSO kinetic equation and higher R^2^ (for Cu^2+^ and Zn^2+^) were obtained for AN carbon, enriched in nitrogen by ammoxidation. As the pseudo-first-order and pseudo-second-order kinetic models do not identify the adsorption diffusion mechanism, the intraparticle diffusion model was tested to define the rate-controlling steps.

Kinetics of adsorption from water solutions includes a few stages [25]:

− transfer of metal ions from the bulk of solution to the interface region,
− transport of metal ions from the interface region to the adsorbent surface (the so-called external diffusion),
− transport of metal ions from the adsorbent surface to the active sites inside the adsorbent (the so-called internal diffusion),
− adsorption of metal ions on the active sites of the adsorbent.

According to this model, if the plot of q versus t^1/2^ gives a straight line passing through the origin of the coordination system, the adsorption process is controlled by the intraparticle diffusion, whereas if the data exhibit multilinear plots, then two or more steps influence the process. In our studies, the plot does not pass through the origin of the coordination system, and hence the intraparticle diffusion is not the rate-limiting step in this process [26,27].

### 3.2. The Effect of pH on Metal Adsorption from One- and Two-Component Solutions

**Figure 2:**
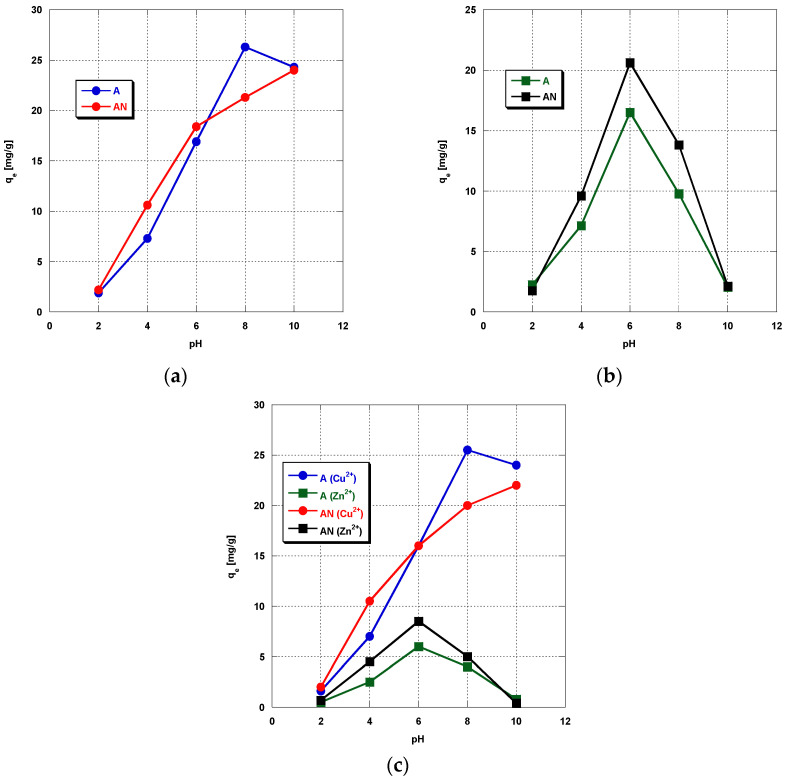
The effect of the solution pH on (**a**) Cu(II) ions’ adsorption, (**b**) Zn(II) ions’ adsorption, and (**c**) simultaneous adsorption of Zn(II) and Cu(II) (mass of sorbent: 0.01 g, heavy metal ions’ concentration: 10 mg/L, time 240 min, shaking speed 180 rpm, temperature: 22 ± 2 °C).

At the next stage of the study, the effect of pH of the solution on the effectiveness of copper(II) and zinc(II) ions adsorption on A and AN was analyzed. As follows from the results presented in Figure 2a–c, the pH of solution has a much greater impact of the adsorption effectiveness than the type of metal or time of sorption. The highest sorption capacities of the carbon adsorbents studied were obtained towards copper(II) ions, irrespective of the type of solutions (single-component or binary). At lower pH (2–6), the sorption capacities were higher for sample AN modified with nitrogen. In the pH range between 8 and 10, higher sorption capacities were noted for unmodified activated carbon, which may be a consequence of the almost twofold higher content of carboxyl groups on the surface [16].

As is commonly known, there are many possibilities for interactions between metal ions and carbonaceous sorbents’ surface, including physical sorption in micro- and mesopores, ion-exchange processes, surface complexes’ formation, or redox reactions with a change in metal valence [28]. Metal ions in water solutions occur in different species that can be adsorbed on the adsorbent surface to different degrees or can precipitate on the adsorbent surface. Therefore, the type of metal species in solution has a significant effect on the adsorption effectiveness. In solutions of pH below 6, the dominant zinc species are Zn^2+^ ions; when pH increases above 6, the concentrations of ZnOH^+^ increase. At pH values higher than 8, precipitation of zinc (II) hydroxide takes place, whereas when the solution pH increases above 10, anionic forms such as Zn(OH)_3_^−^ and Zn(OH)_4_^2−^ are also possible. As far as copper species are concerned, in the solutions of pH close to 6, the dominant species are Cu^2+^ ions, whereas in the pH range between 6 and 10, cationic species like CuOH^+^ and Cu(OH)_2_^2+^ are observed [29]. At higher pH values (7–12), precipitation of copper(II) hydroxide takes place, while at pH above 9, anionic forms, i.e., Cu(OH)_3_^−^ and Cu(OH)_4_^2−^, are also possible. In our experiment, the initial water solutions of pH higher than 6 grew increasingly turbid with increasing pH; moreover, in the neutral and basic environments, a sediment appeared on the bottom of the flask. Many authors who studied the effect of pH on adsorption [30,31] have reported the increase in adsorption of the copper and zinc ions with increasing pH, hence the pH-dependent adsorption. It has also been established that, in strongly acidic solutions, the active sites, that is, carboxyl groups, are protonated, which restricts the adsorption of positively charged metal ions [32]. Moreover, at low pH (<2.5), the surface functional groups of the adsorbent are protonated and the excess of H^+^ ions compete with the metal ions for the active sites on the adsorbent surface, which considerably restricts the adsorption of metals. With increasing pH, the concentration of hydrogen ions in the solution decreases as a result of deprotonation of functional groups, which, at the unchanged concentration of metal ions, has a beneficial effect on sorption effectiveness [28,33].

Changes in pH of the solution also affect the nature of charge on the adsorbent surface. A higher pH can lead to the appearance of negative charge on the adsorbent surface, which enhances the electrostatic attraction between metal cations and the negatively charged carbon surface and leads to increased metal sorption. In the case of the tested activated carbons, the pH_pzc_ value determined by the drift method was 5.9 and 6.4 for the unmodified and N-doped sample, respectively. Thus, higher sorption capacities observed in the case of copper ions at higher pH values of the metal ion solutions may be a consequence of the aforementioned electrostatic interactions. The different tendency of sorption capacity changes in relation to copper and zinc ions depending on the pH of the solution may be a consequence of the different size of their hydrated ions, different ratio of charge to metal ions radius, as well as the different ability to form complexes with functional groups present on the surface of the activated carbons used. However, further research is required to fully explain this issue.

### 3.3. The Effect of Initial Heavy Metal Concentration on Sorption Capacity

**Figure 3:**
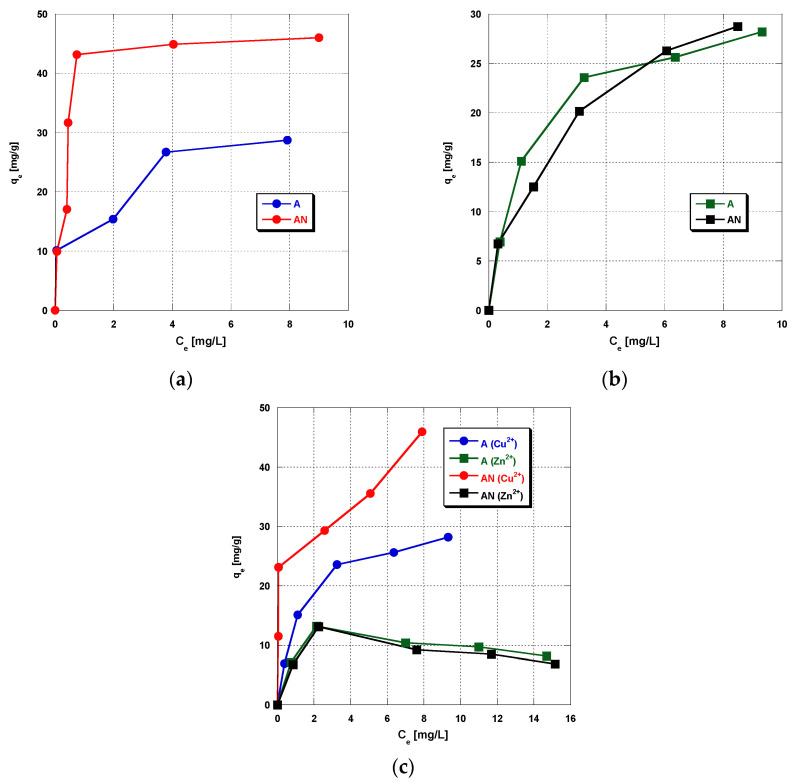
The effect of the initial heavy metal ion concentration on (**a**) Cu(II) ions’ adsorption, (**b**) Zn(II) ions’ adsorption, and (**c**) simultaneous adsorption of Zn(II) and Cu(II) (pH 6, time 240 min, shaking speed 180 rpm, temperature: 22 ± 2 °C).

The results presented in Figure 3a–c are the evidence that each adsorbent material tested is characterized by good sorption abilities towards Zn^2+^ and Cu^2+^ ions, from either single-component or binary solutions, which is probably related to their well-developed surface area and the microporous nature of the structure—the contribution of micropores in the total pore volume is 0.94 and 0.95 for A and AN sample, respectively [15]. The sorption capacity of the activated carbon materials studied towards the metal ions depends on the initial concentrations of Zn^2+^ and Cu^2+^ ions and on the type of carbon modification. According to the results presented in Figure 3a, the AN sample (despite a much smaller specific surface area (by 279 m^2^/g) shows sorption capacity towards copper(II) ions from the single-component solution by about 15 mg/g higher than the unmodified adsorbent, which indicates the beneficial effect of the presence of nitrogen functional groups on the sorption capacity. N-doped activated carbon (containing nitrogen in the form of amines, amides, nitriles or imines, and lactams [16], can potentially bind with copper(II) ions and form surface complexes, and thus increase the efficiency of adsorption [28]. Unfortunately, in the case of zinc(II) ions (Figure 3b). no similar tendency is observed—both types of adsorbent, A and AN, show comparable sorption capacities of about 30 mg Zn^2+^/g_ads_.

The activated carbons obtained show similar adsorption properties towards Cu^2+^, irrespective of the type of solution (mono-component or binary). When adsorption takes place from a binary solution containing Zn^2+^ and Cu^2+^ ions in the same concentrations, the activated carbons obtained show much higher sorption capacities towards copper(II) than towards zinc(II), which is particularly pronounced for the nitrogen-enriched carbon. The two types of carbon adsorbents show similar sorption capacities towards zinc(II) ions and they are about three times smaller than those from the single-component solution. Moreover, the adsorption isotherms have almost the same character for the two types of adsorbents (Figure 3c). The amount of adsorbed Zn^2+^ ions increases with the increasing initial concentrations of the binary solution, then a significant decrease in the amount of adsorbed zinc(II) ions is observed. These results suggest that, at lower concentrations, Zn^2+^ and Cu^2+^ ions can bind to different active centers on the carbon surface or there is an excess of active centers on the adsorbent surface, so very little competition takes place. At higher concentrations of the binary solution, Cu(II) can compete with Zn(II) for binding sites. This competition is likely owing to the higher affinity of the activated carbons to copper than zinc ions, as confirmed by the data shown in Figure 3. Copper ions as more electronegative ones may be removed first, and in consequence of this, zinc(II) ions can only adsorb on the remaining unsaturated active centers present on the activated carbon surface. However, further research (for example, determination of the influence of the concentration of metal ions, the order of adding adsorbates to the system, the presence of other metal ions, or the influence of the ionic strength) is required in order to unambiguously explain this issue.

### 3.4. The Effect of Temperature on Sorption Capacity

**Figure 4:**
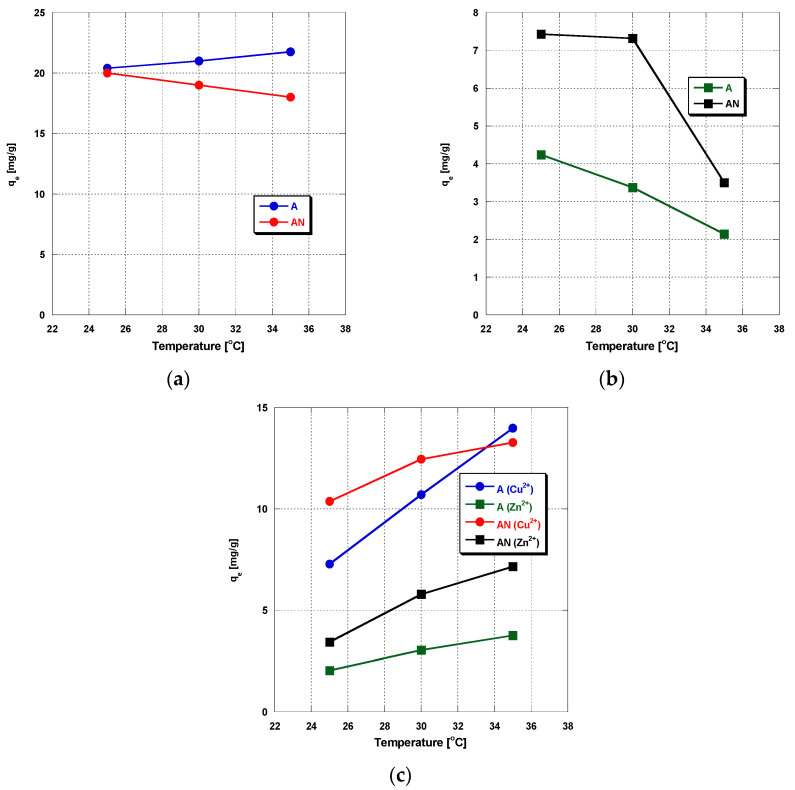
The effect of temperature on (**a**) Cu(II) ions’ adsorption, (**b**) Zn(II) ions’ adsorption, and (**c**) simultaneous adsorption of Zn(II) and Cu(II) (pH: 6, heavy metal ions’ concentration: 10 mg/L, mass of sorbent: 0.01 g, time 240 min, shaking speed 180 rpm).

The results concerning the effect of temperature on the process of metal ions’ adsorption are presented in Figure 4a–c. They indicate that the temperature of adsorption also has some impact on the sorption capacities of the carbon adsorbents studied. When adsorption is carried out from the single-component solutions (Figure 4a,b), the decrease in the sorption capacity with increasing temperature suggests that the reaction between the carbon adsorbents and metal ions was exothermic and the adsorption was mainly based on a physical process that dominates in low temperatures. The decrease in sorption capacity with increasing temperature can also be a result of weakening of bonds between the Cu^2+^ and Zn^2+^ ions and the active sites on the adsorbents. When adsorption is performed from the binary solution, for both carbon adsorbents, an increase in the sorption capacity towards Cu^2+^ and Zn^2+^ ions is observed, which suggests an endothermic nature of the process. Detailed explanation of this problem also needs further study.

### 3.5. Sorption Isotherms Studies

The equilibrium adsorption isotherms of heavy metals onto the investigated carbonaceous sorbents were obtained by examining the relationship between C_e_ and q_e_ at 240 min, at 22 °C, pH 6, and comparing the experimental values of sorption capacity of the sorbents studied with the theoretical sorption capacities determined according to the Langmuir (Equation (5)) and Freundlich (Equation (6)) isotherm equations [22,23]. The model fitting results are shown in Table 4. The Langmuir isotherm model gave the highest correlation coefficient values, showing that the adsorption of Cu^2+^ and Zn^2+^ on activated carbons obtained was described better by this model. Thus, it can be expected that the Cu^2+^ and Zn^2+^ sorption by the adsorbents studied is more likely to be monolayer than heterogeneous surface sorption [34,35]. Additionally, the Langmuir isotherm assumes uniform adsorption energies onto the surface and the absence of interactions among the adsorbed molecules. Using the Freundlich isotherm model for the evaluation of equilibrium parameters, the Freundlich constant (*K_F_*) was determined (Table 5), which was higher for copper(II) ions adsorbed from single-component and binary solutions, for both carbonaceous adsorbents studied. Higher values of K_F_ for the adsorption of copper(II) ions obtained when the process was performed on AN sample, modified by ammoxidation, indicate a higher affinity of Cu^2+^ ions to the nitrogen-enriched sample than to the unmodified sample. For Zn^2+^ ions, the reverse tendency was observed as the Freundlich constant (*K_F_*) was higher for A adsorbent. Another important parameter describing the mechanism of sorption is the *n* constant informing about the character of adsorption. For all adsorbents studied, *n* was much higher than 1, which suggests the physical character of the sorption [1].

According to the data presented in Table 6, the maximum adsorption capacities obtained for the samples studied in this work (especially N-doped activated carbon) are comparable to or higher than the literature values for many adsorbents obtained from different precursors. For example, the adsorption capacity towards copper(II) ions exceeds the results obtained for activated carbons modified by tannic acid [36], sodium diethyl dithiocarbamate immobilization [37], or non-thermal plasma [38], although they were much lower than the results obtained for pre-oxidized and ammoxidized commercial activated carbon (optimum adsorption capacity ~250 mg/g) [39] or carbonaceous nanofibers [40] and crosslinked chitosan [41]. Similar results were obtained for zinc(II) ions. For example, the adsorption capacities are significantly higher than for tannic acid or sodium diethyl dithiocarbamate immobilized activated carbons [36,37] or granular commercial activated carbon [42]; however, they are slightly lower than for the activated carbon from almond husks [43] or dairy manure-derived biochar [44].

### 3.6. SEM, XRD, and FTIR Study of the Activated Carbons before and after Metal Ions’ Adsorption

**Figure 5:**
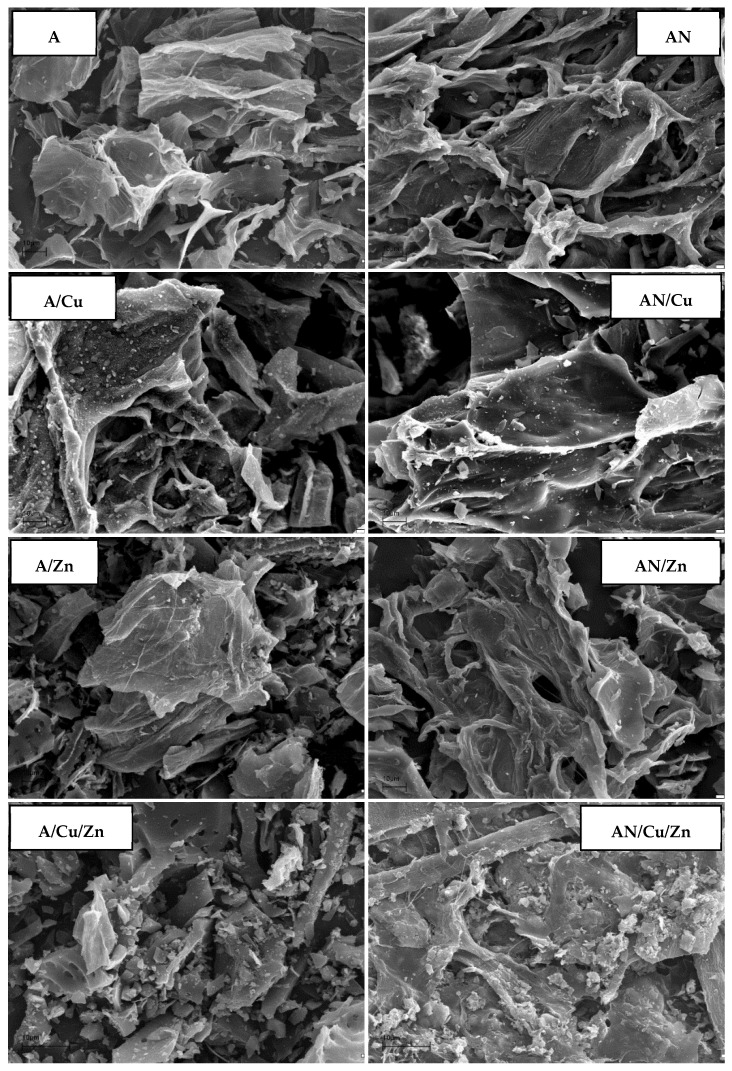
SEM images of the activated carbons before and after Cu(II) and Zn(II) ions’ adsorption (pH: 6, heavy metal ions’ concentration: 10 mg/L, mass of sorbent: 0.01 g, time 240 min, shaking speed 180 rpm).

According to the photomicrographs presented in Figure 5, both activated carbons (A and AN) show very similar morphologies. The structure of the samples consists of numerous pores of various shapes and sizes. The porous systems formed in unmodified and N-doped activated carbons are well-developed, but unevenly distributed. The bright, small fragments visible in the SEM images may indicate the presence of ash (mineral matter) in the structure of the studied materials. As seen, as a result of the adsorption of both metal ions, the morphologies of the samples undergo quite significant changes. Many of the previously open pores have been blocked, and numerous, bright fragments are visible on the surface of the adsorbents, indicating a deposition of adsorbates. This is especially well seen in the case of simultaneous adsorption of copper and zinc ions.

**Figure 6:**
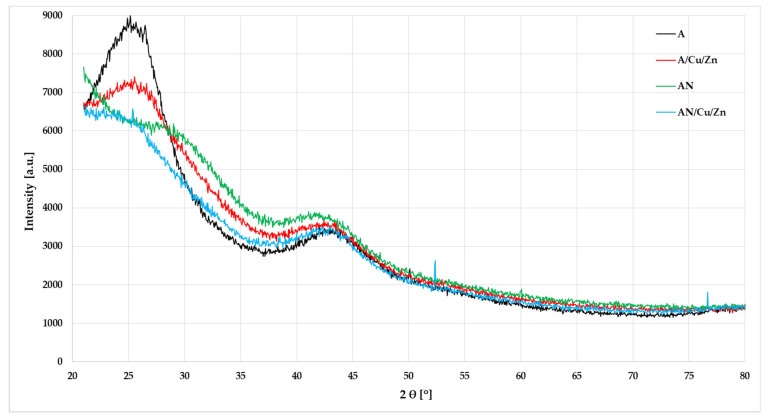
The XRD patterns of the activated carbons before and after Cu(II) and Zn(II) ions’ adsorption (pH: 6, heavy metal ions’ concentration: 10 mg/L, mass of adsorbent: 0.01 g, time 240 min, shaking speed 180 rpm).

**Figure 7:**
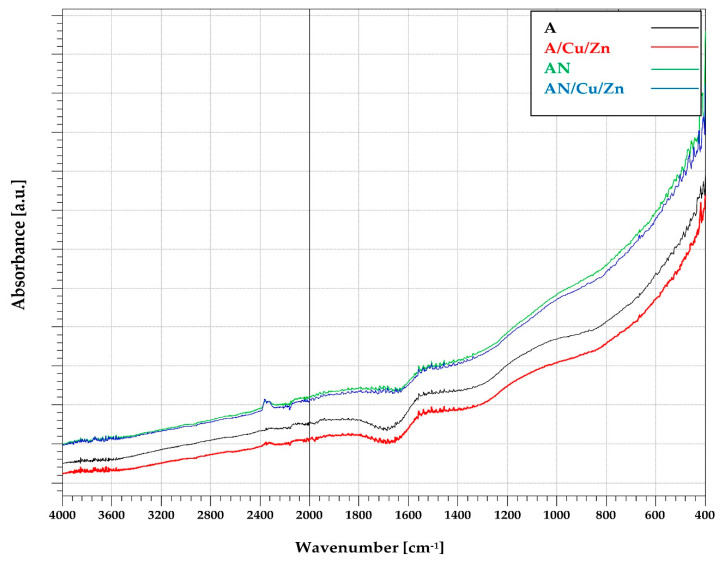
The FTIR spectra of the activated carbons before and after Cu(II) and Zn(II) ions’ adsorption (pH: 6, heavy metal ions’ concentration: 10 mg/L, mass of adsorbent: 0.01 g, time 240 min, shaking speed 180 rpm).

The XRD patterns and FTIR spectra presented in Figure 6 and Figure 7 also indicate slight differences in the structure of A and AN activated carbons. The appearance of a broad diffraction background and the absence of sharp peaks (Figure 6) reveal a predominantly amorphous structure of both activated carbons. There are two very broad diffraction peaks around 2θ at about 25° and 43° in the spectra, corresponding to the diffraction of (002) and (10 0), respectively. However, for the AN sample, the peak observed at 2θ close to 25° is less pronounced. The obtained data show that adsorption of metal ions does not significantly affect the course of XRD patterns. Only in the case of the nitrogen-enriched sample, low-intensity reflections near 25.4, 52.2, and 76.6 were observed, which most probably can be attributed to adsorbed copper or zinc nitrates.

The broad band observed at 1700–1750 cm^−1^ in the ATR-FTIR spectrum of the unmodified sample A (Figure 7) can be attributed to C = O stretching vibration in non-aromatic carboxyl groups. As a result of ammoxidation of the activated carbon, this band disappears. For the nitrogen-enriched sample (AN), only a very small band at 1650 cm^−1^ is observed, which can be ascribed to aromatic skeletal vibrations, as well as to structures containing −C = N− bonds. The course of the ATR-FTIR spectra after the adsorption of Cu(II) and Zn(II) ions does not change (for both the A and AN sample), which may suggest that the adsorption of metal ions is physical and takes place mainly inside the pores of activated carbons.

## 4. Conclusions

The results obtained have shown that the choice of adsorption parameters is very important for the evaluation of sorption abilities of activated carbon adsorbents obtained from brown coal towards heavy metal ions from single-component and binary systems. Irrespective of the type of adsorbent, the optimum time of heavy metal ions’ removal from the model solutions was 60–120 min. With increasing alkalinity of the solution, the effectiveness of adsorption on the materials obtained increased. The best results were obtained at pH 8 for the adsorption of Cu^2+^ ions and pH 6 for that of Zn^2+^ ions. With the increasing temperature of the solution, to a maximum of 35 °C, the effectiveness of adsorption of Cu^2+^ and Zn^2+^ ions from single-component solutions slightly decreases, which suggests an exothermic nature of the process. When the adsorption takes place from the binary solution, the reverse dependence was noted, which indicates an endothermic nature of the process. For the majority of the systems studied, the adsorption is better characterized by the Langmuir isotherm model, in particular when zinc(II) ions are adsorbed from the binary solution, which evidences the formation of an adsorption monolayer on the porous surface of the adsorbents obtained. As far as the kinetics of adsorption is concerned, it was found to be best described by the pseudo-second-order model, which was confirmed by the high values of the respective correlation coefficient. Irrespective of the adsorption parameters (temperature, pH, and initial concentration), the carbonaceous adsorbent subjected to ammoxidation, AN, was more effective in the removal of Cu^2+^ ions from single-component and binary model water solutions. When the adsorption of Zn(II) ions is considered, the modification of carbonaceous materials by ammoxidation gave a beneficial effect only when the process took place from the single-component solution. However, the increase in the effectiveness of sorption relative to that of unmodified sample was much smaller than that observed for copper ions.

## Figures and Tables

**Table 1:** Measurement conditions for analytical technique (F-AAS) used for determinations of Cu and Zn.

Parameter	Cu	Zn
Wavelength [nm]	324.8	213.9
Slit width [nm]	0.7	0.7
Lamp current [mA]	8	5
Lamp mode BGC ^1^	NON	D2
Oxide flow [L/min]	15	15
Fuel flow [L/min]	1.4	1.8
Sample flow rate [mL/min]	4	4
Detection limit (3σ) [mg/L]	0.001	0.001
Determination limit (9σ) [mg/L]	0.003	0.003

^1^ background correction.

**Table 2:** Kinetic parameters for Cu^2+^ adsorption on the activated carbons C_init_ = 10 mg/L.

Sample	Parameters									
	PFO			PSO				IPD		
	k_1_	R^2^	q_cal_	k_2_	h	R^2^	q_cal_	k_i_	C	R^2^
A	0.0099	0.8593	5.39	0.0056	2.989	0.9991	23.15	0.3362	17.19	0.8667
AN	0.0117	0.9552	4.46	0.0082	5.22	0.9997	25.19	0.2867	20.36	0.7277

**Table 3:** Kinetic parameters for Zn^2+^ adsorption on the activated carbons C_init_ = 10 mg/L.

Sample	Parameters									
	PFO			PSO				IPD		
	k_1_	R^2^	q_cal_	k_2_	h	R^2^	q_cal_	k_i_	C	R^2^
A	0.0074	0.5038	1.352	0.144	3.817	0.9927	5.14	0.0963	3.34	0.6945
AN	0.0051	0.2074	0.921	0.0238	1.764	0.9989	8.60	0.1081	6.82	0.6381

**Table 4:** Kinetic parameters for simultaneous Cu^2+^ and Zn^2+^ adsorption on the activated carbons C_init_ = 10 mg/L.

Metal Ions	Parameters									
	PFO			PSO				IPD		
	k_1_	R^2^	q_cal_	k_2_	h	R^2^	q_cal_	k_i_	C	R^2^
A										
Cu^2+^	−0.0004	0.0066	0.855	0.0196	1.17	0.9868	7.72	0.0644	6.467	0.2655
Zn^2+^	−0.0083	0.6963	0.021	−0.087	−0.34	0.9839	1.98	−0.021	2.385	0.1890
AN										
Cu^2+^	0.0101	0.6405	2.313	0.0142	2.13	0.9980	12.25	0.1565	9.596	0.6996
Zn^2+^	0.0108	0.3389	0.506	0.0872	1.70	0.9966	4.42	0.0367	3.835	0.3299

**Table 5:** Langmuir and Freundlich isotherm parameters and correlation coefficients for the adsorption of Cu^2+^ and Zn^2+^ on the activated carbons.

Model	Parameters	A		AN		A Simultaneous AdsorptionCu^2+^ and Zn^2+^		AN Simultaneous AdsorptionCu^2+^ and Zn^2+^	
		Cu^2+^	Zn^2+^	Cu^2+^	Zn^2+^	Cu^2+^	Zn^2+^	Cu^2+^	Zn^2+^
Langmuir	q_0_ [mg/g]	29.59	29.94	47.39	31.55	29.94	8.56	43.48	7.28
	K_L_ [L/mg]	1.94	1.228	4.057	0.828	1.228	−2.41	2.77	−1.776
	R^2^	0.9508	0.9847	0.9953	0.9484	0.9847	0.9821	0.9598	0.9735
Freundlich	K_F_ [mg/g (mg/L)^1/nF^]	17.54	12.20	29.63	11.212	12.20	9.16	27.40	8.97
	n	4.89	2.339	3.25	2.19	2.34	39.06	5.203	42.19
	R^2^	0.8593	0.9310	0.7398	0.9900	0.9310	0.0179	0.7815	0.0114

**Table 6:** Adsorption capacity toward Cu(II) and Zn(II) ions for various carbonaceous materials.

Material	Metal Ion	Maximum Capacity [mg/g]	Article
N-doped activated carbon prepared from brown coal	Cu(II)	47.4	(this study)
aminated commercial activated carbon		~100	[28]
tannic acid immobilized activated carbon		2.23	[36]
sodium diethyl dithiocarbamate (SDDC) immobilised activated carbon		38.0	[37]
non-thermal plasma modified activated carbons		37.6	[38]
pre-oxidised and ammoxidised commercial activated carbon		~250	[39]
carbonaceous nanofibers		204.1	[40]
crosslinked chitosan		200.0	[41]
granular commercial activated carbon		24.5	[42]
amino –factionalized nano-adsorbent		12.4	[45]
activated carbon from walnut shells		28.4	[46]
spirogyra (green alga)		133.0	[47]
N-doped activated carbon prepared from brown coal	Zn(II)	31.5	(this study)
tannic acid immobilized activated carbon		1.23	[36]
sodium diethyl dithiocarbamate (SDDC) immobilised activated carbon		9.9	[37]
granular commercial activated carbon		8.8	[42]
activated carbon from almond husks		35.3	[43]
dairy manure-derived biochar		31.6	[44]
activated carbon from birch sawdust		20.8	[48]
activated carbon from Typha latifolia L		28.6	[49]
activated carbon from date stones		12.2	[50]

## Data Availability

Data are contained within the article.
